# Characterization of *Plasmodium vivax* Proteins in Plasma-Derived Exosomes From Malaria-Infected Liver-Chimeric Humanized Mice

**DOI:** 10.3389/fmicb.2018.01271

**Published:** 2018-06-25

**Authors:** Melisa Gualdrón-López, Erika L. Flannery, Niwat Kangwanrangsan, Vorada Chuenchob, Dietmar Fernandez-Orth, Joan Segui-Barber, Felix Royo, Juan M. Falcón-Pérez, Carmen Fernandez-Becerra, Marcus V. G. Lacerda, Stefan H. I. Kappe, Jetsumon Sattabongkot, Juan R. Gonzalez, Sebastian A. Mikolajczak, Hernando A. del Portillo

**Affiliations:** ^1^Instituto Salud Global, Hospital Clinic—Universitat de Barcelona, Barcelona, Spain; ^2^Institute for Health Sciences Trias I Pujol, Barcelona, Spain; ^3^Center for Infectious Disease Research, Seattle, WA, United States; ^4^Department of Pathobiology, Faculty of Science, Mahidol University, Bangkok, Thailand; ^5^Exosomes Laboratory, CIC bioGUNE, Centro de Investigación Biomédica en Red de Enfermedades Hepáticas y Digestivas (CIBERHD), Derio, Spain; ^6^Metabolomics platform, CIC bioGUNE, CIBERehd, Derio, Spain; ^7^IKERBASKE Basque Foundation for Science, Bilbao, Spain; ^8^Fundação de Medicina Tropical Dr Heitor Vieira Dourado, Manaus, Brazil; ^9^Instituto Leônidas & Maria Deane, Manaus, Brazil; ^10^Mahidol Vivax Research Unit, Faculty of Tropical Medicine, Mahidol University, Bangkok, Thailand; ^11^Catalan Institution for Research and Advanced Studies, Barcelona, Spain

**Keywords:** *Plasmodium vivax*, hypnozoite, exosome, proteomics, biomarker, humanized mice

## Abstract

Exosomes are extracellular vesicles of endocytic origin containing molecular signatures implying the cell of origin; thus, they offer a unique opportunity to discover biomarkers of disease. *Plasmodium vivax*, responsible for more than half of all malaria cases outside Africa, is a major obstacle in the goal of malaria elimination due to the presence of dormant liver stages (hypnozoites), which after the initial infection may reactivate to cause disease. Hypnozoite infection is asymptomatic and there are currently no diagnostic tools to detect their presence. The human liver-chimeric (FRG huHep) mouse is a robust *P. vivax* infection model for exo-erythrocytic development of liver stages, including hypnozoites. We studied the proteome of plasma-derived exosomes isolated from *P. vivax* infected FRG huHep mice with the objective of identifying liver-stage expressed parasite proteins indicative of infection. Proteomic analysis of these exosomes showed the presence of 290 and 234 proteins from mouse and human origin, respectively, including canonical exosomal markers. Human proteins include proteins previously detected in liver-derived exosomes, highlighting the potential of this chimeric mouse model to study plasma exosomes derived unequivocally from human hepatocytes. Noticeably, we identified 17 parasite proteins including enzymes, surface proteins, components of the endocytic pathway and translation machinery, as well as uncharacterized proteins. Western blot analysis validated the presence of human arginase-I and an uncharacterized *P. vivax* protein in plasma-derived exosomes. This study represents a proof-of-principle that plasma-derived exosomes from *P. vivax* infected FRG-huHep mice contain human hepatocyte and *P. vivax* proteins with the potential to unveil biological features of liver infection and identify biomarkers of hypnozoite infection.

## Introduction

*Plasmodium vivax* is a eukaryotic parasite that causes vivax malaria, a disease previously considered to be a benign form of malaria but now recognized to be associated with severe disease and responsible for considerable morbidity and mortality in endemic regions (Mueller et al., 2009; Kevin Baird, 2013). This parasite is widely distributed in tropical and temperate areas, including Ethiopia, Southeast Asia and South America, where 8.5 million clinical cases occur each year, accounting for more than half of all malaria cases outside Africa (World Health Organization, 2017). In 2015, WHO proposed an ambitious global goal that targets the control and elimination of malaria in 35 countries by the year 2030. In that sense, *P. vivax* represents a major barrier to achieve this goal as this species evolved a dormant form called a hypnozoite (Krotoski et al., 1982) that can remain latent for weeks, months or even years in the liver after the initial infection. Hypnozoites can reactivate and cause a blood stage infection and symptoms, called a relapse, which also allows for the possibility of transmission (Krotoski, 1985; Markus, 2011). Importantly, relapses are responsible for the vast majority of cases of vivax malaria in endemic countries where disease burden specifically associated with relapses has been estimated (Betuela et al., 2012; Nacher et al., 2013). Current diagnostic tools are unable to detect asymptomatic patients harboring hypnozoites in their liver, implying the existence of a large reservoir of parasites. This is not only detrimental for people suffering the symptoms of relapsing malaria, but represents a major obstacle toward malaria elimination. Without a sensible diagnostic tool detecting asymptomatic hypnozoites carriers, mosquitos feeding in these individuals will continue to spread *P. vivax*.

The advances in humanized mouse models to study human malaria infections have created opportunities to study host-parasite interactions *in vivo*. More precisely, several humanized mouse models with a human chimeric liver have been developed (SCID Alb-uPA, FRG huHep, TkNOG, and the AFC8-hu HSC/Hep) from which two of them (SCID Alb-uPA and FRG huHep) have been successfully used to model extra-erythrocytic infections in *P. falciparum* (Morosan et al., 2006; Vaughan et al., 2012). An extended overview of humanized mice models adapted to malaria parasites have been reviewed previously (Kaushansky et al., 2014). Importantly, a major breakthrough in the field of *P. vivax* research was achieved by the development of the FRG huHep chimeric mouse and its implementation as a robust model for the development of pre-erythrocytic stages of *P. vivax*, notably the growth and reactivation of hypnozoites. This model allows research into the biological properties of this liver stage and the use of the model to test the activity of potential radical cure drugs (Mikolajczak et al., 2015).

Exosomes are 30–120 nm extracellular vesicles (EVs) of endocytic origin secreted by eukaryotic cells and present in virtually all-biological fluids (Yáñez-Mó et al., 2015). These nanovesicles initially described in reticulocytes as a garbage disposal mechanism during the terminal differentiation of reticulocytes to erythrocytes (Pan and Johnstone, 1983) are currently recognized by their remarkable role in intercellular communication, and their potential as novel therapeutic agents and biomarkers of disease (Théry et al., 2002; Gho and Lee, 2017). Exosomes are formed by invaginations of late endosomes, a process that leads to the engulfment of cytoplasm and specific molecular targets, forming vesicles contained inside multivesicular bodies (MVB) that display lipids and proteins at their external surface and enclose a unique luminal material. These vesicles are released to the extracellular space after fusion of MVB with the plasma membrane. Remarkably, the unique properties of exosomes, including a great stability in circulation, easy detection in complex biological fluids and a molecular content that can be considered a “liquid biopsy,” support their use as excellent biomarkers of disease (Simpson et al., 2009; Revenfeld et al., 2014; Théry, 2015; Nedaeinia et al., 2016; Bautista-López et al., 2017).

Importantly, exosomes and microvesicles have been reported to be involved in intercellular communication, modulation of the immune response, and in the regulation of vascular function in malaria (Sampaio et al., 2017). A pioneer work, described the presence of parasite proteins in exosomes isolated from peripheral blood of *P. yoelii* infected mice (Martin-Jaular et al., 2011). Later, other studies also showed that EVs secreted by *P. falciparum* infected red blood cells (RBC) deliver DNA to other infected cells promoting differentiation to sexual forms, a step required for transmission to mosquitos (Regev-Rudzki et al., 2013). In addition, EVs were also found capable of communicating with host cells modulating the immune response of macrophages and neutrophils (Mantel et al., 2013). In line with these findings, is the recent report that showed the presence of small RNA and genomic DNA of *P. falciparum* in exosome-like vesicles involved in the activation of intracellular DNA sensors in human monocytes (Sisquella et al., 2017). Furthermore, it was reported that EVs derived from *P. falciparum*-infected RBCs contain a functional RNA interference machinery that modulates the expression of important genes in targeted endothelial cells leading to alterations in the physiological properties of endothelial tissue correlating with the pathophysiological features of falciparum malaria (Mantel et al., 2016). In addition, this regulation mechanism seems also to modulate parasites gene expression in targeted infected cells (Wang et al., 2017). Together, all these evidences support the role of EVs, including exosomes, as important mediators of multiple functions in the context of malaria infections. Importantly, all these studies has focus on EVs secreted by blood stages forms of *Plasmodium* spp. parasites while nothing is yet know regarding EVs secreted by liver stage forms.

In this study, we have employed a proteomics approach to explore the possibility of identifying parasite proteins from pre-erythrocytic liver stages associated with exosomes secreted by *P. vivax* infected FRG huHep mice; thus, opening the path for searching for biomarkers of hypnozoite infection in this liver-chimeric model.

## Methods

### *P. vivax* FRG HuHep mouse infections

All animal procedures were conducted in accordance with and approved by the Center for Infectious Disease Research Institutional Animal Care and Use Committee (IACUC). The Center for Infectious Disease Research IACUC adheres to the NIH Office of Laboratory Animal Welfare standards (OLAW welfare assurance #A3640-01).

Mice were infected with *P. vivax* sporozoites as previously described (Mikolajczak et al., 2015). The experimental design was based in two settings: A first experimental infection (EI1) was completed using six female FRG huHep mice by intravenous injection of 1 million sporozoites. Infected mice were euthanized 8 days post infection (dpi), exsanguinated and livers harvested. Four uninfected FRG huHep mice were used as controls. The objective of this experimental infection was to compare the proteomic composition of exosomes during two different conditions (infection vs. non-infection). A second experimental infection (EI2) was completed and female mice were euthanized 8 (*n* = 4), 10 (*n* = 4), 16 (*n* = 3), and 21 (*n* = 1) dpi (Figure S1). Mice euthanized at day 10 received an intraperitoneal injection of human reticulocytes as previously described (Mikolajczak et al., 2015). This setting pursue to compare the proteome of exosomes at different infection time-points in which different liver-stage forms of *P. vivax* and exosomal cargo are expected. Infections were verified either by fluorescence microscopy or qRT-PCR. At each indicated dpi, livers were harvested and immunofluorescence analysis was conducted to characterize parasite liver burden by quantification of *P. vivax* liver schizonts and hypnozoites (Data Sheet S1).

### Blood collection and plasma separation

Animals were euthanized and exsanguinated by cardiac puncture. Whole blood from individual animals was collected in tubes using a syringe preloaded with 0.1 mL of 250U/mL heparin. Samples were processed within 30 min by centrifugation at 2,000 × g for 5 min at 4°C to remove platelets and platelet-derived vesicles. Plasma samples were immediately transferred into a clean tube and frozen at −80°C.

### Exosomes purification

Exosomes were isolated from plasma samples of uninfected and *P. vivax* infected FRG huHep mice by size-exclusion chromatography (SEC) following our own standard methodologies (de Menezes-Neto et al., 2015). Briefly, varying aliquots of plasma (0.3–0.5 mL) were thawed on ice and processed by centrifugation at 2,000 x g for 10 min at 4°C. For exosome purification, plasma collected from infected mice from EI1 was processed individually. For mice in EI2, as plasma volumes were < 0.3 mL, samples from 2 mice were pooled as shown in Figure S1. Plasma supernatant was loaded on the top of commercial qEV Sepharose 2B columns (10 ml) (iZON Sciences) pre-equilibrated with PBS. Fifteen fractions of 500 μl were collected immediately after sample loading and analyzed or frozen at −80°C. Protein concentration was determined by measuring absorbance at 280 nm with a Nanodrop (Thermo Scientific, San Diego, CA).

### Bead-based flow cytometry

Phenotypic characterization of isolated exosomes was done by detection of the exosomal marker CD5L in a bead-based flow cytometry assay. This protein was found to be consistently associated to plasma-derived exosomes in human samples (de Menezes-Neto et al., 2015). Concisely, 50 μl of SEC fractions was coupled to Aldehyde/Sulfate Latex Beads, 4% w/v, 4 μm (Invitrogen) by incubation for 15 min with agitation. Coupled beads were then blocked by incubation overnight with 1 ml of BCB Buffer [(PBS 1X/BSA 0.1% /NaN3 0.01% (both from Sigma-Aldrich)] in a rotation device. Beads were further centrifuged at 2,000 x g for 10 min, supernatant removed and pelleted beads were re-suspended in 100 μl of BCB buffer. 45 μl of bead suspension was incubated with anti-CD5L antibodies (Abcam: ab45408) at 1/200 or IgG isotype control (Santa Cruz: SC-3888) at 1/1000 for 30 min at 4°C in a round bottom plastic microplate. Note that anti-CD5L recognize both mouse and human CD5L antibody. After washing, samples were incubated with a rabbit secondary-antibody conjugated to Alexa 488 (Invitrogen: A11008) at 1/1000 dilution for 30 min at 4°C protected from light. After two wash steps, beads were re-suspended in 100 μl of PBS and analyzed by flow cytometry using a BD FACSVerse (BD Biosciences) machine. Median Fluorescence Intensity (MFI) and bead count data were obtained using FlowJo v.X Software (TreeStar). As control for specificity, we have incubated SEC fraction 7 or 8 in the presence of a rabbit isotype IgG antibody and secondary-antibody Alexa 488 (C1: Exos+iso+2°).

### Nanoparticle track analysis

Size distribution and particle concentration was determined by Nanoparticle Track Analysis (NTA) in a NanoSight LM10-12 instrument (Malvern Instruments Ltd, Malvern, UK) as previously reported in (de Menezes-Neto et al., 2015) using the NTA software (version 3.2).

### Cryo-electronmicroscopy (cryo-EM)

SEC fractions containing high (F8) and low (F12) CD5L MFI values were analyzed by cryo-EM to estimate size and morphology of isolated vesicles as described elsewhere (Montaner-Tarbes et al., 2016). Briefly, 10 μl of SEC fractions was diluted 1/5 in 0.22 μm filtered PBS and laid on a Quantifoil® 1.2/1.3 TEM grid, blotted to a thin film and plunged into liquid ethane-N_2_(l) in the Leica EM CPC cryoworkstation (Leica, Wetzlar, Germany). Grids were transferred to a 626 Gatan cryoholder and maintained at −179°C. Samples were analyzed with a Jeol JEM 2011 transmission electron microscope (Jeol, Tokyo, Japan) at an accelerating voltage of 200 kV. Images were recorded on a Gatan Ultrascan 2000 cooled charge-coupled device (CCD) camera with the Digital Micrograph software package (Gatan, Pleasanton, CA). Vesicle diameter was quantified using ImageJ (NIH) where pixels were calibrated to nanometers.

### Cloning, expression and purification of recombinant truncated PVX110940

The protein sequence of PVX110940 was retrieved from Plasmodium Genomic Database (PlasmoDB) (http://plasmodb.org/). B-cell linear epitopes prediction was done using the Bebipred algorithm (Jespersen et al., 2017) in the portal of Immune Epitope Database and Analysis Resource [(IEDB) http://www.iedb.org/]. Based on Bebipred score, a region of 407 aa corresponding to amino acids 261 and 667 was amplified from genomic DNA of the *P. vivax* Sal-I strain using the primers: PVX110940-Tr-F: 5′-TAAGAATGCGGCCGCGAGGATGTGCTGCCAAGTGT-3′ and PVX110940-Tr-R: 5′- TTACTCGAGTCATTCATCCTCCGCTTCATCCTC-3′. PCR amplified fragment was digested with NotI and XhoI (Thermo scientific) and directly ligated into the pIVEX1.4-GST vector from which N-terminal GST-tagged fusion proteins are expressed (Rui et al., 2011). After cloning, the construct was analyzed by DNA sequencing. GST-PV110940-tr *in vitro* protein expression was done in a commercial wheat germ cell-free system (Biotechrabbit) accordingly to manufactures instructions. Briefly, 4 μg of plasmidic DNA from two positive clones was used as a template in 50 μl of *in vitro* transcription/translation reaction. GST-PVX110940-Tr recombinant protein was purified by affinity chromatography on GST spin trap columns (GE healthcare) and eluted with 10 mM glutathione in 50 mM Tris-HCl pH 8.0. Purified protein was desalted against sterile PBS in a 10 kDa Amicon devise (Millipore). Bradford assay (Biorad) was used to quantify protein concentration. Protein production was confirmed by SDS-PAGE and western blot analysis using anti-GST primary antibody (1/5000) (Invitrogen: A5800) and 680LT fluorescent conjugated rabbit secondary antibody (Licor 92568021) (Li-Cor Biosciences, Lincoln, NE, USA)].

### Polyclonal antibodies generation

The immunization procedure was approved by the Animal Experimentation Ethics Committee of the “Hospital Universitari Germans Trias I Pujol” under the protocol number DAAM907. Two 6-week-old female BALB/c mice were immunized by subcutaneous injection using 10 μg of purified recombinant GST-PVX110940-Tr in combination with aluminum hydroxide. Two consecutive boosts were done at intervals of 21 days after which animals were euthanized and bled by cardiac puncture. Blood was incubated during 2 h at RT to promote coagulation and subsequently sera were collected from the supernatant. Immunoreactivity was evaluated against purified GST-PVX110940-tr recombinant protein by western blotting.

### Western blot analysis

Protein samples were heated at 95°C for 5 min and separated on a 10–12% SDS-PAGE, transferred on to Hybond-C nitrocellulose membrane (Amersham) and blocked in blocking buffer (1 X PBS, 0.1% Tween-20, 5% milk powder) overnight. After washes, blots were incubated for 1 h with primary antibodies [rabbit anti-GST (1:5000, Invitrogen: A5800), mouse anti-GST-PVX110940-Tr (1/500 or 1/50), rabbit anti-arginase I (1/1000) (Genentex: GTX109242)] in antibodies buffer (1X PBS, 0.1% Tween-20, 1% milk powder). Subsequently, the blots were washed and incubated for 1 h with the Li-Cor IRDye-labeled secondary antibodies IRDye 680RD goat anti-rabbit (925-68021) (1/20.000) and IRDye 800CW goat anti-mouse (925-32210) (1/15.000) (Li-Cor Biosciences, Lincoln, NE, USA). Blots were scanned and analyzed with the Odyssey quantitative western blot near-infrared system (Li-Cor Biosciences, Lincoln, NE, USA) using default settings, with the exception of 700-laser intensity, which was set up at 3.

### Exosome solubilization and protein digestion for LC-MS/MS

Hundred micro liters of SEC fractions F7, F8, F9, F10 were pooled and designated as exosomal-enriched fractions (ExEFs). Similarly, 100 μl of SEC fractions F5, F6, F11, F12 were pooled and designated as microvesicles-enriched fraction (MvEF). ExEF and MvEF were mixed with equal volume of RIPA buffer [50 mM Tris pH 8, 150 mM NaCl, 1 mM EDTA, 0.5 % NP-40, 10 mM MgCl_2_, 0.5 mM DTT, 1:100 protease inhibitors (Thermo Scientific)], incubated at 70°C and further sonicated for 10 min with cycles of 30 s at the highest intensity (Bioruptor-Diagenode) to ensure exosomal membrane disruption. Samples were centrifuged at 16,000 x g during 15 min at 4°C and supernatant recovered. Proteins were then precipitated with cold acetone [1/6 ratio (v/v)] O/N at −20°C and recovered after subsequent centrifugation at 16,000 x g during 15 min at 4°C. Precipitated proteins were resuspended in 30 μl 6M urea and further reduced in 10 mM dithiothreitol (DTT; Sigma) for 1 h at 37°C and alkylated with 55 mM of iodoacetoamine (IAM, Sigma) during 30 min at RT. Samples were brought to 2M urea and digested with a concentration of Lys-C (Wako) corresponding to 10% (μg) of the digested sample, overnight at 37°C. Subsequently, samples were further diluted to 1M urea and digested for 12 h with a concentration of trypsine (Promega) corresponding to 10% (μg) of the digested sample. Finally, samples were desalted on MicroSpin C18 columns (The Nest Group), evaporated to dryness and dissolved in 0.1% formic acid.

### Mass spectrometry

Approximately 1 μg of each sample was analyzed using liquid chromatography (nanoLCULTRA-EKSIGENT) followed by mass spectrometry on a 90 min gradient in the Orbitrap Fusion Lumos (Thermo Fisher Scientific). As a quality control, BSA controls were digested in parallel and ran between each sample to avoid carryover and assess the instrument performance. The Mass spectrometry proteomic data have been deposited to the ProteomeXchange Consortium via PRIDE (Vizcaíno et al., 2016) partner repository with an dataset identifier PXD008945.

### Protein identification

Raw data files were analyzed with the search algorithm Mascot v2.5.1 (http://www.matrixscience.com) on the Proteome DiscovererTM Software V2.0 (Thermo Scientific) using three protein databases: UniProt *P. vivax* (31,374 entries), UniProt human (20,133 entries) and UniProt mouse databases (16,832 entries), downloaded on April 28th, 2017. Peptides have been filtered based on: (i) minimum peptide length of 7; (ii) maximum false discovery rate (FDR) for peptides and proteins of 1%; (iii) minimum peptides per protein of 1 and minimum unique peptides per protein of 1; (iv) minimum score for modified peptides was set to 40; (v) main search error of 4 ppm. Mouse and human proteins were accepted if more than 2 unique peptides were identified with a false discovery rate < 1% and assigned with the category of Master protein by Proteome DiscovererTM software. In the case of proteins identified by 1 unique peptide, a criterion of its presence in at least 2 samples was applied. Assigned contaminants, keratins and abundant proteins plasma reported in (de Menezes-Neto et al., 2015) were removed. Human and mouse proteins were compared to the public extracellular vesicle Vesiclepedia protein database (http://www.microvesicles.org/) and intersections were represented in the FunRich Software (Pathan et al., 2015). For *P. vivax* protein identification, a different criterion was applied, accepting proteins identified with 1 unique peptide, FDR < 1%, category of Master Protein by Proteome Discoverer software after confirming individual high quality spectrum. *P. vivax* proteins UniProt entries were used to retrieve protein sequences and blasted to the PlasmoDB against *P. vivax* proteins in order to identify and assign the corresponding gene name in the SalI and P01 strains. Redundant entries were filtered out from the final list of proteins.

Gene ontology enrichment of human proteins for all categories was done with the Database for Annotation, Visualization, and Integrated Discovery (David 6.8) (Huang et al., 2009).

### Statistical analysis

Protein abundance data was estimated from the average peak area of the three top peptides detected by Proteome DiscovererTM Software v2.0 (Thermo Scientific). Briefly, data were normalized using quantile normalization. Then, data were log2-transformed to hold normality assumption that is required in downstream analyses. Statistical analyses in both comparisons Infected vs. Control and ExEF vs. MvEF was performed by using lineal or censored regression models. Censored data appear in those samples having average peak area values below 10^5^ correspond to values not detected. Protein data were analyzed using linear models implemented in limma Bioconductor package (Ritchie et al., 2015). Those proteins having censored data in < 50% of samples were analyzed using censored regression, implemented in censreg R package (Henningsen et al., 2017), as proposed here (Helsel, 2005).

## Results

### Isolation of plasma-derived exosomes from human liver-chimeric FRG huHep mice

**Figure 1:**
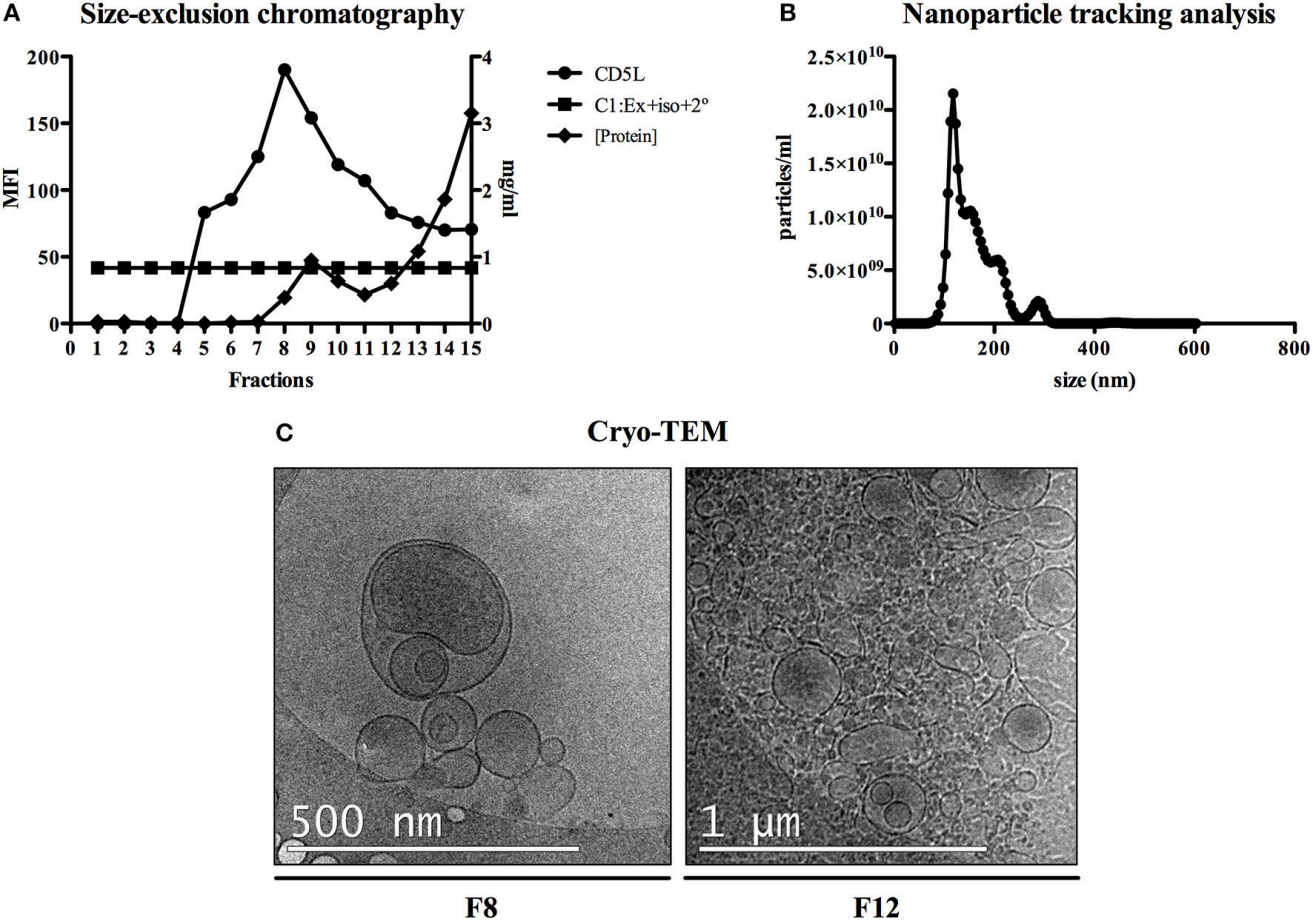
Exosomes purification and characterization. **(A)** SEC fractions of plasma-derived exosomes from *P. vivax* infected FRG huHep mice from EI1 and EI2 were analyzed in a flow cytometry bead-based assay for the presence of exosomal marker CD5L. Mean fluorescence intensity (MFI) of anti-CD5L and control antibodies (rabbit-isotype) as well as protein concentration was assayed for each fraction and a representative analysis for a single sample is shown. **(B)** NTA profile (size [nm] vs. concentration [particles/ml]) of the fraction with the highest CD5L MFI (F8) shows enrichment in nanoparticles around 120 nm. **(C)** Cryo-EM of F8 confirms the presence of abundant, round nanovesicles measuring 100–150 nm. SEC fraction 12 (F12) identified aggregates and nanovesicles of diverse sizes.

To initially explore the protein content of plasma-derived exosomes in FRG huHep mice during *P. vivax* infection, we analyzed individual plasma samples from two groups of mice: a control group corresponding to uninfected mice and a group of infected mice euthanized 8 dpi (EI1). Further, and taking into account the liver infection kinetics previously established by Mikolajczak et al. (2015), we analyzed plasma samples of *P. vivax* infected mice after 8, 10, 16, and 21 dpi in order to get insight into possible changes in the protein content of plasma-derived exosomes associated with the infection by specific parasite liver stages (EI2). Plasma samples from both experiments were processed for exosome isolation by SEC. SEC fractions were evaluated in a bead-based flow cytometry assay for the detection of CD5L, an exosomal marker identified in MS studies of exosomes isolated from plasma (de Menezes-Neto et al., 2015). The distribution of CD5L signal throughout SEC fractions showed a standard and reproducible profile with the majority of exosomes eluting between fractions 7 and 10, constituting the exosome-enriched fractions (ExEFs) (Figure 1A and Figures S2A,B). Similar results were obtained with CD9 and CD81, other exosomal markers (data not shown). To further characterize the isolated exosomes in the ExEFs, we performed NTA of individual and pooled fractions 7–10 to determine the size and concentration of particles within these fractions and conducted cryo-electronmicroscopy (Cryo-TEM) specifically of fraction 8 (F8) to characterize vesicle size and morphology. Moreover, for comparative purposes, we also performed Cryo-TEM of F12, enriched in microvesicles and soluble proteins (MvEF). NTA showed vesicles with a mode diameter of 117 nm and a concentration of 2.67 x 10^11^ particles/ml (Figure 1B). Cryo-TEM demonstrated highly pure, round vesicles free of protein aggregate clumps and 42% of vesicles in F8 were between 50 and 100 nm in diameter (Figure 1C and data not shown). In contrast, an aggregated and complex material was observed in SEC distal fraction 12 where microvesicles, other particles and soluble plasma proteins are expected (Figure 1C).

### Proteomic analysis of plasma-derived exosomes from *P. vivax* infected FRG huHep mice: the human component

**Figure 2:**
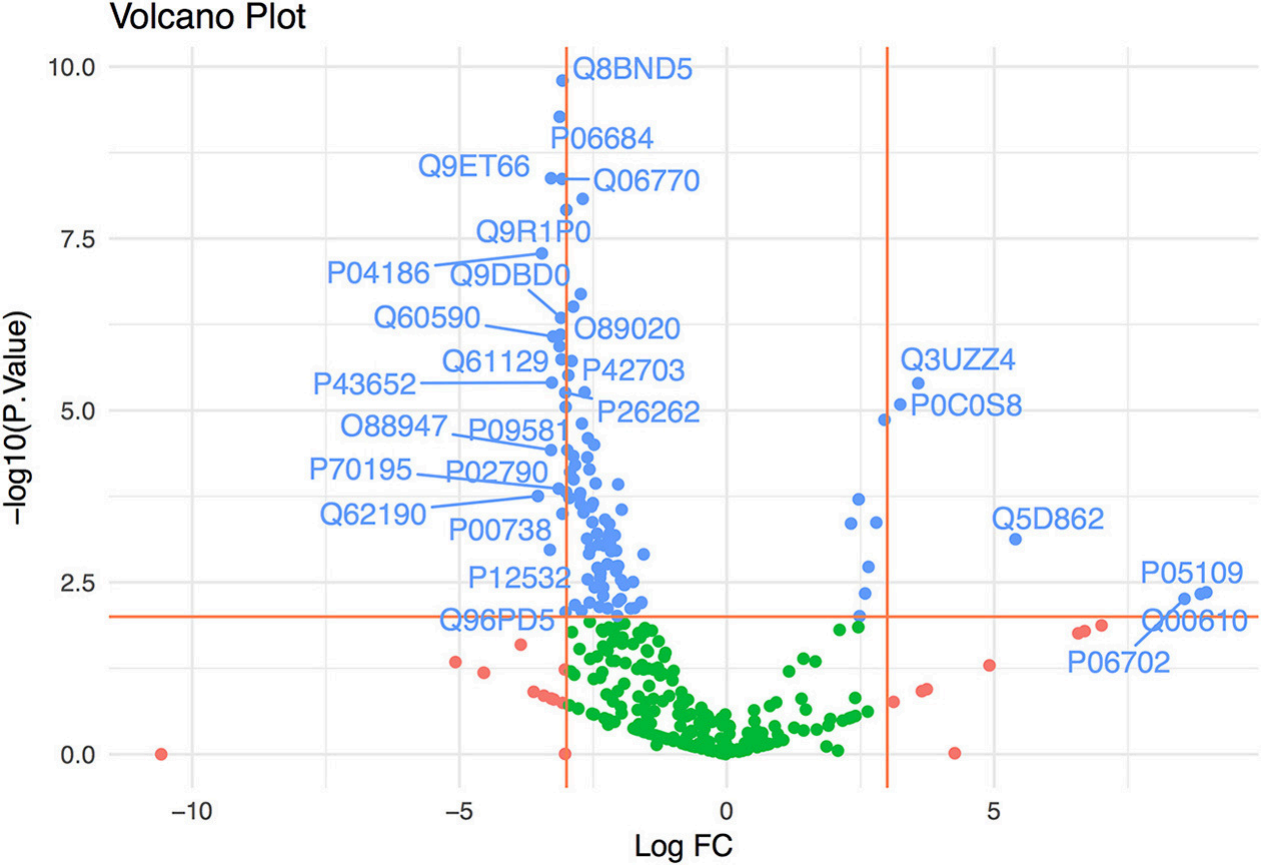
Exosome-enriched fractions (ExEFs) are depleted of contaminating plasma proteins. The log fold-change (FC) in abundance of human proteins detected by LC-MS/MS in the ExEFs and MvEFs of *P. vivax* infected FRG huHep mice from EI1 were compared. The horizontal line represents *p* = 0.01 and vertical lines correspond to a 3-log fold increase or decrease in abundance. Green points represent *p* > 0.01 & FC between [−3,3], blue points represent *p* < 0.01 and red points represent *p* > 0.01 & FC >3 or FC < −3. Proteins with *p* < 0.01 and FC>3 or FC < -3 are labeled. Supporting data are shown in Data Sheet S3.

We determined the proteome of ExEFs and searched for peptide spectrum matches against human, mouse and *P. vivax* protein databases. Data from EI1 and EI2 identified 234 human proteins, 290 mouse proteins and 17 *P. vivax* proteins (Data Sheet S2) collectively. To further demonstrate the ExEFs were mostly depleted of abundant contaminating plasma proteins, thus avoiding this major confounder in MS analysis, we performed a statistical comparison of the human identified proteins enriched in the ExEFs and those enriched in the MvEF from EI1, using a statistical method based on censured and lineal data (Helsel, 2011; Henningsen et al., 2017). We have centered our statistical analysis in samples from EI1 because the number of replicate samples in this setting give us sufficient statistical power to perform a robust comparison. As shown in Figure 2 and Data Sheet S3, most plasma proteins were significantly depleted in ExEFs, as estimated from the fold change of the average peak area of the 3 top peptides for each identified protein in the ExEF compared to the MvEF. Altogether, these results strongly suggest that plasma-derived exosomes from *P. vivax* infected FRG huHep mice are efficiently isolated by SEC and that abundant plasma proteins, a large confounding effect in MS analysis (de Menezes-Neto et al., 2015), were largely depleted by this single-standing methodology.

**Figure 3:**
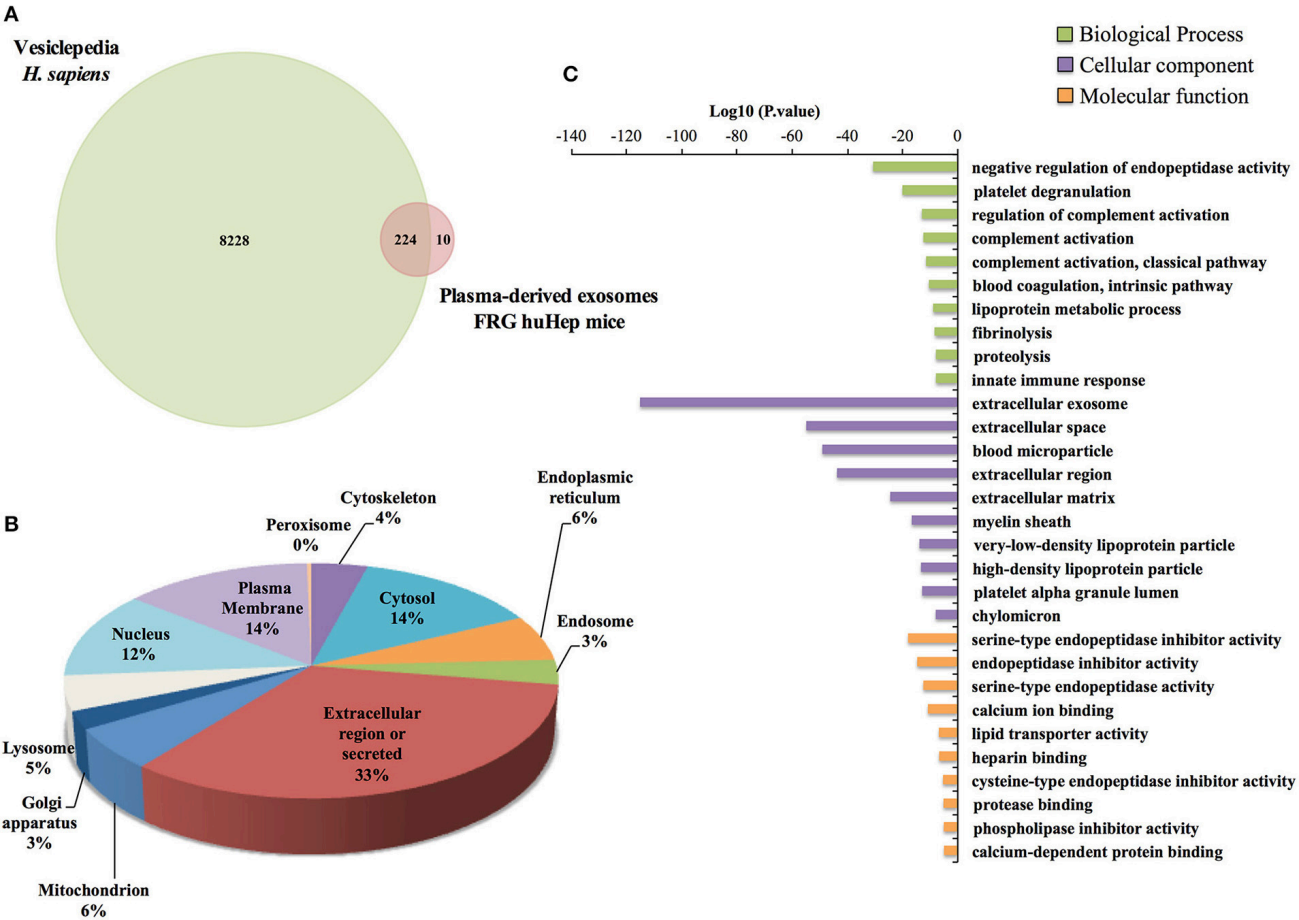
Human Proteome of FRG huHep plasma-derived exosomes. **(A)** Global analysis of all human proteins identified in EI1 and EI2 shows that of the 234 human proteins identified in the ExEFs from uninfected and *P. viv*ax infected FRG huHep mice, 224 are reported in Vesiclepedia, a Web-Based repository of extracellular vesicle cargo proteins. **(B)** Subcellular localization distribution of 234 human proteins groups identified in plasma derived-exosomes from uninfected and *P. vivax* infected mice. Subcellular compartment was assigned based on GO terms in the UniProt database. **(C)** Functional enrichment analysis of the top ten more significant Gene ontology (GO) terms of human proteins group enriched in exosomes derived from plasma of uninfected and *P. vivax* infected FRG huHep mice (*P-value* 0.001). Bar plot shows biological process, cellular component, and molecular function GO categories.

Because human hepatocytes are the only human cells present in these chimeric mice, we initially analyzed the protein content of human hepatocyte-derived exosomes. Global comparison of the human proteins identified compared with proteins listed in Vesiclepedia, a public repository of extracellular vesicle proteins, showed that 96% of human proteins associated with exosomes in this mouse model have been previously reported as associated with extracellular vesicles (Figure 3A), including classical exosomal markers such as clathrin heavy chain 1, annexin A2, glyceraldehyde-3-phosphate dehydrogenase, moesin, and Ras-related Rap-1b-like proteins. Next, subcellular localization of the identified human proteins was assigned based on GO terms retrieved from UniProt and the frequency of proteins found in each subcellular compartment. This analysis showed that a considerable percentage corresponds to the extracellular region (33%), while the two other more abundant compartments were the plasma membrane and cytosol (Figure 3B). To gain insight into the function of the human proteins, a GO enrichment analysis was performed (Figure 3C and Data Sheet S4). As expected, the Cellular component category showed a significant enrichment of proteins associated with extracellular exosomes and blood microparticles. The Biological processes category showed enrichment of proteins involved in the negative regulation of endopeptidase activity and platelet degranulation whereas Molecular function was enriched for endopeptidase activities and its inhibition.

Several proteomes of hepatocyte-derived exosomes has been previously published (Conde-Vancells et al., 2008; Zhao et al., 2014; Jia et al., 2017). These studies have employed *in vitro* cultured rat hepatocytes and the immortalized hepatocytes HepAD38 and Huh7 cell lines as sources of exosomes isolated from the culture supernatant. In order to explore the correlation between our data and that of those publications, we compared the human proteins associated with exosomes in the *P. vivax* infected and uninfected FRG huHep mice with these previously published proteomes. Importantly, 106 out of 234 proteins identified in our study (45%), have been previously reported in at least 1 proteome whereas 128 (55%) were newly identified liver-exosome cargo proteins. As expected from the different isolation methods and analysis, a different number of common proteins was found on individual comparisons of each report with our data set (Data Sheet S5).

**Figure 4:**
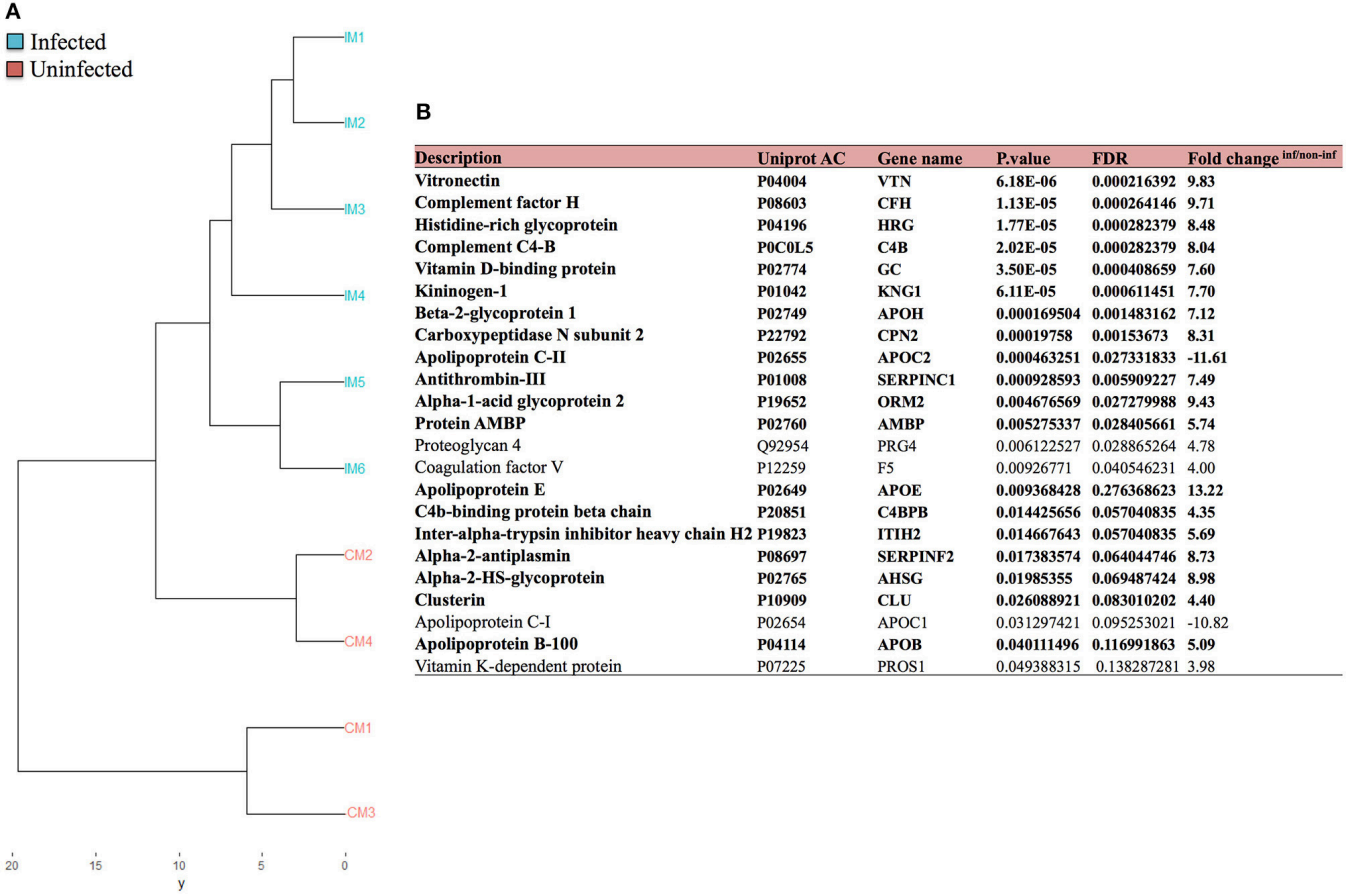
Comparison of human liver proteins from *P. vivax* infected FRG KO huHep mice with plasma proteins from patients in natural infections. **(A)** Dendogram depicting the results of a statistical comparison (supervised and lineal methods) between the human proteins identified by LC-MS/MS-based proteomics in enriched exosomes derived from plasma of *P. vivax* infected and uninfected FRG huHep mice from EI1. IM-1 to IM-6 (Infected mice), CM-1 to CM-4 (uninfected mice). Supporting data are shown in Data Sheet 3. **(B)** Twenty-three human proteins found to be significantly over-represented in infected FRG KO huHep mice (*P*-value < 0.05, FDR adjusted) were compared with the proteome of total plasma proteins from *P. vivax* patients (Ray et al., 2016). Nineteen proteins (in bold) were detected in both datasets.

In order to identify human liver proteins associated with exosomes during *P. vivax* infection, we performed a statistical comparison of human proteins identified in infected animals versus uninfected control mice. This analysis showed that several proteins identified in the ExEFs were differentially associated with *P. vivax* infections (*P*-value < 0.05). We classified the samples in a dendogram taking into account the significant proteins expression values from the comparison infected vs. uninfected in FRG huHep mice. The dendogram separated the samples according to infection (IM; CM) although some variability was present (Figure 4A and Data Sheet S3). The list of proteins used for the dendogram is detailed in the Figure 4B. The table informs not only about the protein but also about the *p*-value (corrected by FDR) and fold change obtained in the comparison. Interestingly, when we compared these proteins to a recently published proteome of total plasma proteins from *P. vivax* patients (Ray et al., 2016), we found that 19 out of 23 of the statistically significant human proteins associated with *P. vivax* infection in the mice were also present in the plasma of *P. vivax* infected patients (Figure 4B, bold). These results indicate that *P. vivax* infection in FRG huHep mice recreates the dynamic changes that occur in the plasma protein content during natural *P. vivax* infections in human.

### Proteomic analysis of plasma-derived exosomes from *P. vivax* infected FRG huHep mice: the parasite component

MS analysis revealed the presence of 17 *P. vivax* proteins in ExEFs (Table 1). Importantly, this dataset excluded false positive *P. vivax* proteins identified in identical peptide spectrum matches of ExEFs from uninfected FRG huHep mice. Among the identified proteins we found a putative chaperon HSP70 (PVX_099315), the merozoite surface protein MSP3.1 (PVX_097670), proteins involved in DNA replication, chromatin structure and ribosome biogenesis [DNA helicase (PVX_099345), Histone H2A (PVX_095190), and 50S ribosomal L24 (PVX_123825)]; the putative enzymes methyltransferase (PVX_000890), diacylglycerol kinase (PVX_116900) and carbamoyl phosphate synthetase (PVX_122240); a putative protein from the parasite endocytic pathway: clathrin coat assembly protein AP50 (PVX_118455), two integral membrane proteins identified as a putative potassium channel (PVX_123990) and a putative member of the mitochondrial carrier protein family (PVX_115395); as well as five uncharacterized hypothetical proteins (PVX_090185, PVX_110940, PVX_115100, PVX_119280, PVX_080600) and a putative “moonlight” oocyst capsule protein (PVX_095215).

**Table 1:** *P. vivax* proteins identified in plasma-derived exosomes from FRG huHep infected mice.

**Protein**	**UniProt AC**	**PlasmoDB entry**	**Experimental infection**	**Detection**			
				**D8**	**D10**	**D16**	**D21**
ATP-dependent DNA helicase Q1, putative	A5K6Q0	PVX_099345	EI1	*			
Methyltransferase, putative	A0A0J9SJ43	PVX_000890	EI1	*			
Mitochondrial carrier protein, putative	A0A0J9TA28	PVX_115395	EI1				
Hypothetical protein, conserved	A0A0J9U1I1	PVX_090185	EI1	*			
Merozoite surface protein 3 (MSP3.1)	A0A0J9V216	PVX_097670	EI1	*			
Heat shock protein 70, putative	A0A0J9TFN8	PVX_099315	EI2	*	*	*	
Hypothetical protein, conserved	A5KDU3	PVX_110940	EI2				
Histone H2A, putative	A0A0J9TEH1	PVX_095190	EI2		*		
Potassium channel, putative	A5K072	PVX_123990	EI2	*			
Diacylglycerol kinase, putative	A0A0J9VER0	PVX_116900	EI2		*		
Hypothetical protein, conserved	A0A0J9TSI7	PVX_115100	EI2				
Clathrin coat assembly protein AP50, putative	A5K403	PVX_118455	EI2				
Hypothetical protein, conserved	A0A0J9SCT6	PVX_119280	EI2				
Carbamoyl phosphate synthetase, putative	A0A0J9SP67	PVX_122240	EI2				
Oocyst capsule protein, putative	A0A0J9SW43	PVX_095215	EI2				
Hypothetical protein, conserved	A0A0J9SBG1	PVX_080600	EI2				
50S ribosomal protein L24, putative	A0A0J9TNT0	PVX_123825	EI2	*			

17 P. vivax proteins were identified in the exosome-enriched SEC fractions (ExEF) of infected FRG huHep mice. Proteins identified in the samples from experimental infection 1 and 2 are depicted. Gray boxes indicate protein presence.

* *Proteins also identified in the microvesicle-enriched SEC fractions (MvEF—Table 2) at the indicated days post infection. Of note, high quality spectrum of individual peptides were manually inspected. Extended mass spectrometry data is found in the Data Sheet S2*.

We compared the distribution of these proteins at the different infection time points, with the objective of identifying parasite proteins secreted from infected cells that could be associated with a specific *P. vivax* liver stage. Interestingly, we found two proteins present at all infection time points: heat shock protein 70 (PVX_099315) and the putative histone H2A (PVX_095190) while the majority of the proteins showed a variable distribution (Table 1). Some proteins were detected at specific time points, notably three proteins (PVX_118455, PVX_119280, PVX_122240) were uniquely present in the ExEFs from day 16 and 21 post-infection. At these time points during infection the majority of replicating schizonts present in the liver have egressed and the remaining parasites are non-replicating hypnozoites. Because liver schizonts at day 8 contain tens of thousands of merozoites, the hypnozoite signal is easily muted in these samples.

In order to maximize the possibility of identifying other *P. vivax* proteins secreted either as soluble factors or associated with other subtypes of extracellular vesicles, we also included in our analysis the MvEFs. Interestingly, we identified peptides corresponding to 20 parasite proteins (Table 2). These included several putative membrane associated proteins from the PIR multigene family [variable surface protein Vir12 (PVX_106220), an unspecified product orthologue to a PIR protein of strain PVP01 (PVX_241290), VIR protein (PVX_101503) and a variable surface protein Vir9-related (PVX_096935)]; two isoforms of the Merozoite surface protein 3 family [MSP3.5 (PVX_097690) and MSP3.10 (PVX_097720)]; a putative reticulocyte binding protein 1b (PVX_098582), a putative THO complex subunit 2 (PVX_101385), a protein involved in transcription/export of mRNAs; putative enzymes like the ATP-dependent acyl-CoA synthetase (PVX_002785) and M18 aspartyl aminopeptidase (PVX_087090); a member of the ribosomal 26S complex [26 complex proteasome regulatory subunit p55, putative (PVX_001760)] and 9 hypothetical proteins of unknown function (PVX_111420, PVX_089245, PVX_087720, PVX_092720, PVX_090990, PVX_082938, PVX_124005, PVX_095185, PVX_117060).

**Table 2:** *Plasmodium vivax* proteins identified in the microvesicles-enriched SEC fractions (MvEF) of plasma-derived exosomes from FRG huHep infected mice.

**Protein**	**UniProt AC**	**PlasmoDB entry**	**Experimental infection**	**Detection**			
				**D8**	**D10**	**D16**	**D21**
Variable surface protein Vir12, putative	A0A0J9U104	PVX_106220	EI1				
Hypothetical protein	A5KE32	PVX_111420	EI1				
Hypothetical protein, conserved	A0A0J9SJ40	PVX_089245	EI1				
Reticulocyte binding protein 1b	A0A0J9SH34	PVX_098582	EI1				
Hypothetical protein, conserved	A5KA42	PVX_087720	EI1/EI2				
Hypothetical protein, conserved	A0A0J9TD53	PVX_092720	EI1/EI2				
THO complex subunit 2, putative	A0A0J9S679	PVX_101385	EI1				
Hypothetical protein, conserved	A0A0J9SCY7	PVX_090990	EI1/EI2				
Hypothetical protein, conserved	A0A0J9SQK8	PVX_082938	EI1				
Hypothetical protein, conserved	A0A0J9SNU2	PVX_124005	EI2				
ATP-dependent acyl-CoA synthetase, putative	A0A0J9T1B9	PVX_002785	EI2				
M18 aspartyl aminopeptidase, putative	A0A0J9TXR1	PVX_087090	EI2				
Merozoite surface protein 3 (MSP3.5)	A0A0J9U2T8	PVX_097690	EI2				
PIR protein_unspecified product	A0A0J9TLW5	PVX_241290	EI2				
26S proteasome regulatory subunit p55, putative	A0A0J9SG98	PVX_001760	EI2				
Hypothetical protein, conserved	A0A0J9SW37	PVX_095185	EI2				
Hypothetical protein, conserved	A5K376	PVX_117060	EI2				
Merozoite surface protein 3 (MSP3.10)	A0A0J9SAF3	PVX_097720	EI2				
PIR protein_variable surface protein Vir9-related	A0A0J9SF54	PVX_096935	EI2				
PIR protein_ VIR protein	A0A0J9T8Z3	PVX_101503	EI2		

*20 P. vivax proteins were identified in the MvEFs of infected FRG huHep mice. Proteins identified in the samples from experimental infection 1 and 2 are depicted. Gray boxes indicate protein presence at the indicated days post infection. Of note, high quality spectrum of individual peptides were manually inspected. Extended mass spectrometry data is found in the Data Sheet S2*.

### Validation of detected human and parasite proteins

**Figure 5:**
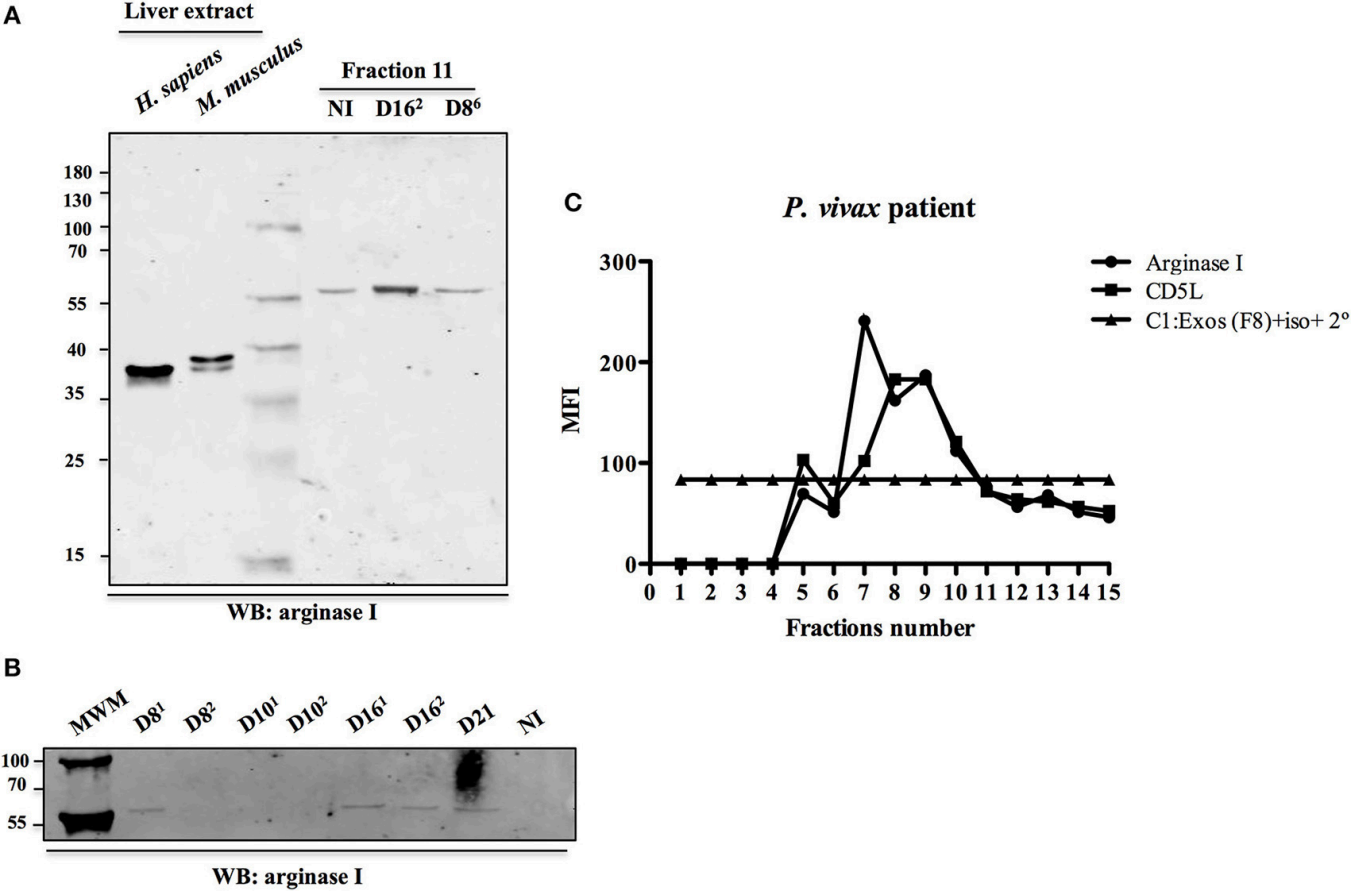
Validation of arginase I as an exosomal-associated protein. **(A)** Commercial anti-arginase I antibodies were used to analyze the presence of arginase I in SEC fraction 11 from *P. vivax* infected at 8 and 16 dpi (EI1: D8^6^ and EI2: D16^2^) and uninfected (NI) FRG huHep mice. These samples correspond to mice where arginase I was identified by LC-MS/MS. An equal volume of each fraction was loaded on the gel. Ten micro grams of liver extract from mouse and human were used as positive controls. **(B)** Western blot analysis of ExEFs purified from *P. vivax* infected FRG huHep mice from EI2 on days 8, 10, 16, and 21 post-infection. An equal volume of CD5L-highest fraction was loaded on the gel. **(C)** Flow cytometry bead-based assay showing arginase I detection in exosomal fractions isolated from plasma of a *P. vivax* patient. This sample correspond to an unidentified stored plasma of the biobank from the FMT-HVD. Mean fluorescence intensity (MFI) of anti-arginase and control antibodies (rabbit-isotype) was assayed for each fraction. CD5L was used as plasma exosomal marker.

We identified in the ExEFs the human enzyme arginase I (Data Sheet S2), a liver protein that converts L-arginine into ornithine, a reaction involved in the polyamine synthesis pathway and in the regulation of nitric oxide metabolism. We got an interest in this enzyme because it was found to be associated to the external side of hepatocyte-derived exosomes from rat origin where it is involved in blood metabolome remodeling and in modulation of endothelium function (Royo et al., 2017) and therefore could be an interesting exosomal protein for validation. Western blot analysis, using a commercial anti-arginase I antibody, validated the presence of this enzyme in exosomal preparations from *P. vivax* infected and uninfected FRG huHep mice (Figures 5A,B). Surprisingly, we detected a band migrating as a protein with an apparent molecular weight of 59 kDa, suggesting an increase of 20 kDa with regard to its predicted molecular weight. Whether this increase is due to a posttranslational modification, caused by the interaction of arginase I with exosomes, is currently under study. Interestingly, we were able to detect in a flow cytometry bead-based assay, an arginase I positive signal in SEC fractions of plasma samples obtained from a *P. vivax* infected patient. The profile followed the signal of the exosomal marker CD5L (Figure 5C), suggesting the presence of an arginase I positive exosome population in the plasma of infected patients.

**Figure 6:**
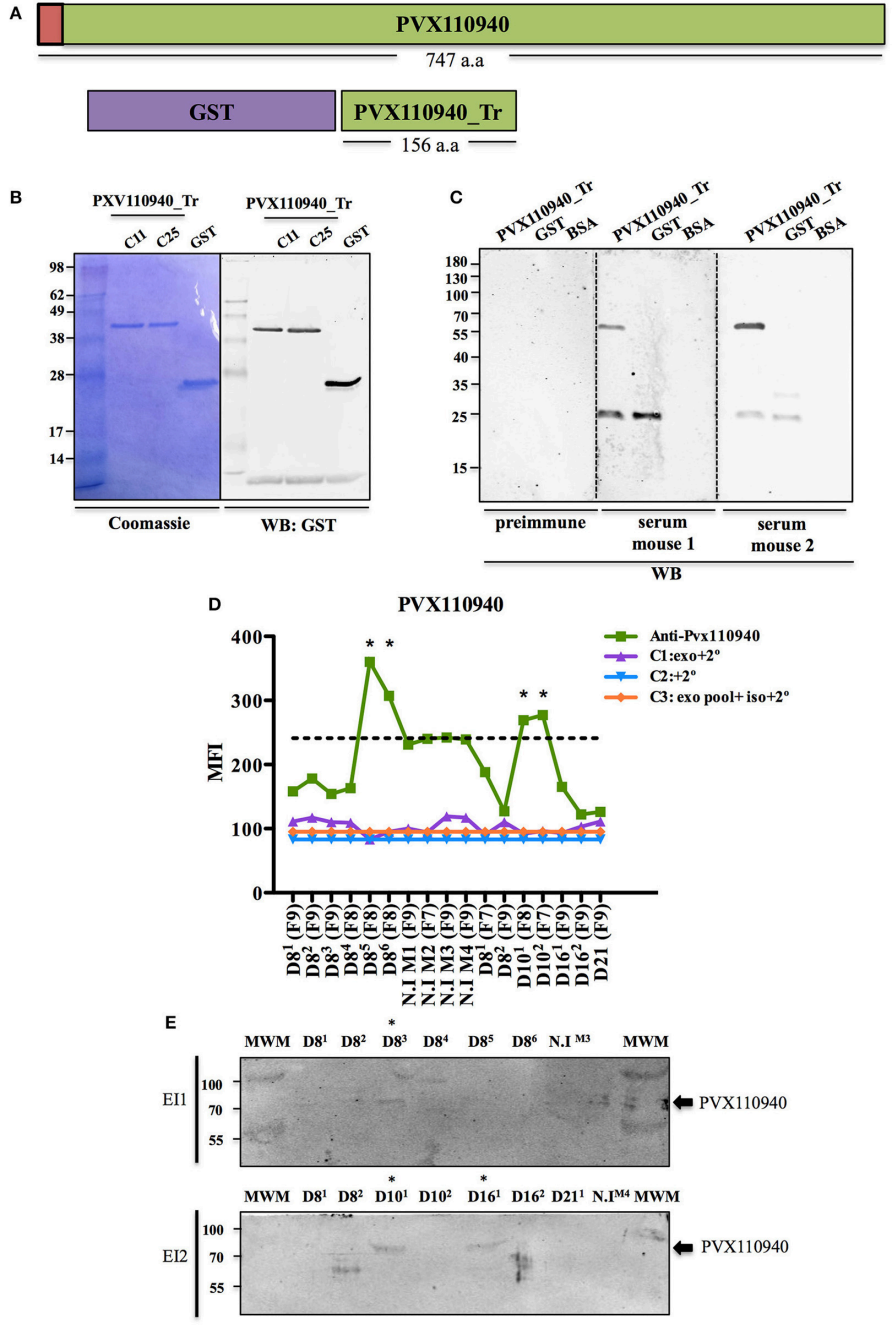
Validation of PVX110940 in plasma-derived exosomes from *P. vivax* infected FRG huHep mice. **(A)** Schematic representation of full-length PVX110940 protein and a recombinant GST-fusion truncated (GST-PVX110940-Tr) version containing a predicted B-cell antigenic region of 156 amino acids. Red square represents a predicted N-terminal signal peptide. **(B)** GST-PV110940-Tr was produced from two *E. coli* clones (C11 and C25) in a wheat germ cell-free system and affinity-purified products resolved on SDS-PAGE. Western blot analysis using monoclonal anti-GST antibodies. **(C)** Recombinant GST-PVX110940-Tr was used to immunize mice and produce polyclonal antibodies. Western blot analysis shows recognition of GST-PVX110940-Tr purified recombinant protein and purified GST. **(D)** Flow cytometry bead-based assay showing PVX110940 detection in ExEF from plasma of *P. vivax* infected FRG huHep mice from EI1 and EI2. Beads were incubated with the highest-CD5L SEC fraction of each sample and used for detection of PVX110940 using the polyclonal antibodies raised in mice. Mean fluorescence intensity (MFI) of PVX110940 and controls was assayed. Specificity controls were as follows: C1: Highest-CD5L SEC fraction of each sample incubated with Alexa-488 mouse secondary antibody. C2: Pool of CD5L highest fraction of all samples incubated with isotype rabbit antibody and Alexa-488 mouse secondary antibody. Stars indicate the samples were positive detection was observed over the background signal observed in uninfected mice (Dashed line). **(E)** Western blot analysis of PVX-110940- in isolated exosomes (CD5L-highest SEC fraction) derived from plasma of *P. vivax* infected FRG huHep mice from EI1 and EI2. Hundred micro liters of CD5L-highest fraction were blotted. Membranes were incubated with 1/50 of polyclonal anti-PVX110940 antibody. ^*^Samples where a positive signal at the expected molecular size (84 kDa) is observed.

To validate the presence of parasite proteins in plasma-derived exosomes of *P. vivax* infected FRG huHep mice, we decided to target the parasite protein identified with the highest number of unique peptides in the ExEFs, PVX_110940. It corresponds to a hypothetical conserved protein of 84 kDa with orthologues in *P. cynomolgi, P. knowlesi, P. fragile*, and *P. inui* and with a predicted signal peptide at its N-terminal end (Figure 6A). Of interest, this gene is located at exactly the same genomic locus as the *P. falciparum* liver stage antigen 1; yet, sequence similarity analysis precluded to include the falciparum gene as truly syntenic. We amplified a fragment of PVX_110940 corresponding to an antigenic region predicted to be a linear epitope of B-cells (Figure 6A) and cloned it using the expression vector pIVEX-GST (Rui et al., 2011). After *in vitro* transcription/translation, a GST-recombinant protein containing a truncated 17 kDa region of the predicted B-cell epitope was purified and shown to specifically react with an anti-GST antibody by western blot analysis (Figure 6B). The purified recombinant protein was used to raise polyclonal antibodies in mice. Western blot analysis using these antibodies demonstrated that this antiserum efficiently recognized the purified recombinant protein (Figure 6C), as well as ExEFs both, in cytometry bead-based assays and western blot (Figures 6D,E respectively). These complementary results indicates the presence of PVX110940 in several samples, notably sample 10^1^ from EI2, where the proteomic analysis identified the protein with 3 unique peptides. All together, these data show that plasma-derived exosomes isolated from *P. vivax* infected FRG HuHep mice contain parasite proteins.

## Discussion

Here, we present the first mass spectrometry (MS)-based proteomics analysis of plasma-derived exosomes from a human liver-chimeric mouse infected with pre-erythrocytic stages, including hypnozoites, of *P. vivax*. Since the discovery of hypnozoites (Krotoski et al., 1982), little progress has been made to decipher biological aspects of this parasite liver stage and the nature of its latency. This is due to the obvious limitations imposed by the inaccessibility to study liver samples from *P. vivax* patients. However, progress has been achieved since the implementation of *in vitro* cultures of *P. cynomolgi* in non-human primate hepatocytes, a relapsing monkey parasite (Dembélé et al., 2014) and *P. vivax* in human hepatocytes (Sattabongkot et al., 2006; Chattopadhyay et al., 2010; Ng et al., 2015), successfully showing the development and reactivation of hypnozoites. These systems are currently being used in transcriptomic studies (Cubi et al., 2017) and hypnozoite-specific drug screening platforms. The recent report of an *in vivo* mouse model where pre-erythrocytic liver stages of *P vivax* can develop and progress to the blood stages, represents an unprecedented opportunity to explore the possibilities of identification of a biomarker of liver stages, notably hypnozoites.

The recent expansion of research in the EV, including exosome, field has put in the spotlight the potential of these vesicles as biomarkers in a large variety of diseases, ranging from infectious diseases, neurodegenerative disorders, and diabetes, to different types of cancer (Simpson et al., 2009; Melo et al., 2015; Thompson et al., 2016; Bautista-López et al., 2017; Garcia-Contreras et al., 2017). Exosomes present in biological fluids are currently being profiled to detect not only particular disease biomarkers, but also molecules that are prognostic of the response to drug treatment and the evolution of some diseases (Simpson et al., 2009).

In this work, we have shown that human hepatocytes engrafted in the liver of chimeric FRG huHep mice represent an appropriate system to obtain hepatocyte derived-exosomes secreted in the bloodstream. The purification approach based on SEC, enabled us to obtain purified exosomes-like vesicles, as was demonstrated by their molecular and structural characterization (Figures 1A,C). Moreover, we could statistically show the depletion of a high number of abundant plasma proteins from ExEFs and the respective enrichment of proteins previously detected in exosomes (Figure 2). Thus, size-exclusion chromatography, as shown elsewhere (de Menezes-Neto et al., 2015), offers a single-standing methodology for isolation and MS characterization of proteins mainly associated with plasma-derived exosomes.

*P. vivax* infection causes alterations in the plasma/serum protein profile as was recently demonstrated in a quantitative proteomic analysis of *P. vivax* patients (Ray et al., 2016). The authors claimed that these changes reflect the pathophysiological changes that occur in the human host during the different stages of vivax malaria infection. Our results indicate that the *in vivo* model of FRG huHep mice recreates those changes as 83% of the human proteins found to be significantly expressed in infected FRG huHep mice (Figure 4) were found to be altered in the plasma of patients during natural infection. Importantly, these also indicate that in natural infection, the liver secretes exosomes with this protein cargo.

Among the identified hepatocyte-exosomal proteins, we found arginase I, a cytosolic enzyme that is mainly synthesized in liver cells and that catalyzes the last step of the urea cycle, converting the amino acid arginine into L-ornithine. This enzymatic reaction also provides the initial substrate to polyamine biosynthesis, a pathway proved to be essential for *P. falciparum* development in liver cells (Meireles et al., 2017). In addition, this enzyme also regulates the nitric oxide (NO) concentration in some cells due to its competition for arginine with the nitric oxide synthase, modulating in this way the synthesis of the key immunomodulatory molecule, NO (Munder, 2009). The identification and validation of arginase I in ExEFs from uninfected and infected mice indicates that this protein could be a candidate liver-specific exosomal marker in this model.

Our group and others have already identified *Plasmodium* spp proteins associated with microvesicles and exosomes by proteomic approaches (Mantel et al., 2013; Martín-Jaular et al., 2016). In both cases, EVs have been obtained from culture supernatant of infected *P. falciparum* and *P. yoelii* infected RBCs, respectively. Both proteomes show the presence of proteins exposed in the RBC membrane, proteins involved in the invasion process and some metabolic enzymes. Another recent study, found that microparticles isolated from the plasma of malaria patients contain parasite proteins including cytoskeleton components, enzymes, surface exposed and heat shock proteins (Antwi-Baffour et al., 2016). The proteins associated with plasma-derived exosomes from *P. vivax* infected FRG KO huHep mice showed low coincidence with those previous reports, probably reflecting the different cell types from which they originate. Only, HSP70, a classical EV marker in all organisms studied so far (Simpson et al., 2009), was found to be common to all malaria EV proteomes until now published, pointing out a selective-sorting mechanism of EVs and/or exosome secretion in *Plasmodium* parasites. The validation of the hypothetical conserved protein PVX_110940 in exosomal fractions of *P. vivax* infected FRG huHep mice (Figure 6), indicates that, in principle, the parasite proteins identified by mass spectrometry could be detected by immunodetection techniques. This is of particular interest in light of the feasibility of detecting hypnozoite specific proteins associated to plasma-derived exosomes. The fact that we identified three proteins uniquely present in ExEFs isolated from FRG huHep plasma samples, after 16 and 21 days of *P. vivax* infection, suggests that these proteins could originate from hypnozoite-infected hepatocytes. Whether these proteins are potential biomarkers of hypnozoite infection is currently under investigation.

In summary, we have demonstrated that the *P. vivax* infected FRG huHep liver chimeric mouse is a unique system for the proteomic profiling of exosomes derived from human infected hepatocytes secreted into the bloodstream. Moreover, our work represents a proof-of-principle that parasite proteins associated with plasma-derived exosomes can be identified by mass-spectrometry in this model, opening the path toward the discovery of hypnozoite biomarkers. Lastly, the identification of human hepatocyte-specific surface biomarkers should facilitate immunocapturing and detection of exosomes in plasma from truly relapsing patients.

## Author contributions

MG-L and JS-B performed exosome purification and LC/MS. MG-L, EF, DF-O, JS-B, CF-B, JG, JS, SM, and HP designed experiments and analyzed the data. NK, EF, SM, and VC performed mouse infections. SK, ML, FR, and JF-P contributed materials. MG-L, EF, and HP drafted the manuscript. All the authors revised the manuscript critically and consent to its publication.

### Conflict of interest statement

The authors declare that the research was conducted in the absence of any commercial or financial relationships that could be construed as a potential conflict of interest.

## Supplementary material

Figure S1 Experimental *P. vivax* infection of FRG huHep mice. **(A)** Six FRG huHep mice were infected with 1 million *P. vivax* sporozoites. Infected mice were euthanized 8 days post-infection (dpi) and blood samples collected. Four uninfected control mice were also euthanized and blood samples collected. **(B)** Twelve FRG huHep mice were infected with *P. vivax* sporozoites and euthanized 8 dpi (four mice), 10 dpi (four mice), 16 dpi (three mice), and 21 dpi (one mouse). After plasma was collected from individual mice, plasma samples from two mice were pooled to generate 2 plasma samples 8 dpi, 2 samples 10 dpi, and 2 samples 16 dpi (1 sample pooled from 2 mice and 1 sample from a single mouse).

Figure S2 Molecular characterization of SEC fractions was done through flow cytometry bead-based analysis of the CD5L marker. **(A)** Experimental infection 1 (EI1). Isolation of exosomes from the plasma of *P. vivax* infected FRG HuHep mice was performed 8 days post infection (dpi). There were 6 infected mice (M1-M6) and 4 uninfected mice (M1-M4). **(B)** Experimental infection 2 (EI2). Isolation of exosomes from plasma of *P. vivax* infected FRG HuHep mice at 8 (2 mice: M1-M2), 10 (2 mice: M1-M2), 16 (2 mice: M1-M2), and 21 (1 mouse) dpi. Images show that CD5L-positive-exosomes were enriched in fractions 7−10. Based on this profile, an exosomal enriched fraction (ExEF) and a plasma-microvesicles enriched fraction (MvEF) were made by pooling fractions F7-F8-F9-F10 (black dashed square) and fractions F5-F6-F11-F12 (red dashed square), respectively. Blue circles: CD5L signal, Purple squares: negative control (Fractions+rabbit-isotype+Alexa488-2^a^ antibody, Black diamonds: Protein concentration.

Data Sheet S1 Characterization of *P. vivax* infection in liver tissue from FRG huHep infected mice.

Data Sheet S2 Mass spectrometry data of *H. sapiens, Mus musculus* and *P. vivax* proteins identified in ExEFs.

Data Sheet S3 Statistical analysis of *H. sapiens* proteins identified in the ExEFs and MvEFs and in the ExEFs of infected and uninfected mice from Experimental infection 1.

Data Sheet S4 Gene ontology enrichment analysis of *H. sapiens* proteins identified in the ExEFs of FRG huHep *P. vivax* infected mice.

Data Sheet S5 Global comparison of *H. sapiens* proteins identified in the ExEFs of *P. vivax* infected FRG huHep mice with the core proteins identified in the previously published proteomes of hepatocytes-derived exosomes.
