# Supplementary figures and images for: Characterization of Plasmodium vivax Proteins in Plasma-Derived Exosomes From Malaria-Infected Liver-Chimeric Humanized Mice
Source: Front Microbiol. 2018 Jun 25;9:1271. doi: 10.3389/fmicb.2018.01271 (PMC6026661; doi:10.3389/fmicb.2018.01271)

### Experimental infection 1

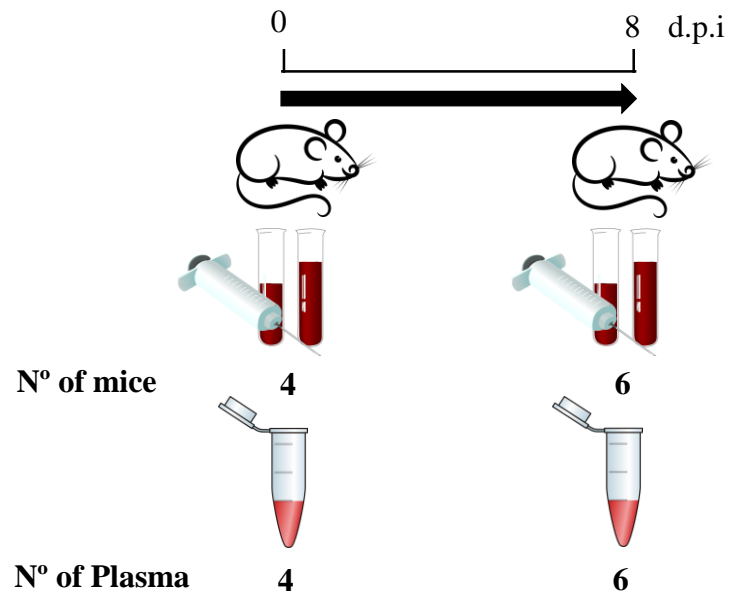

### Experimental infection 2

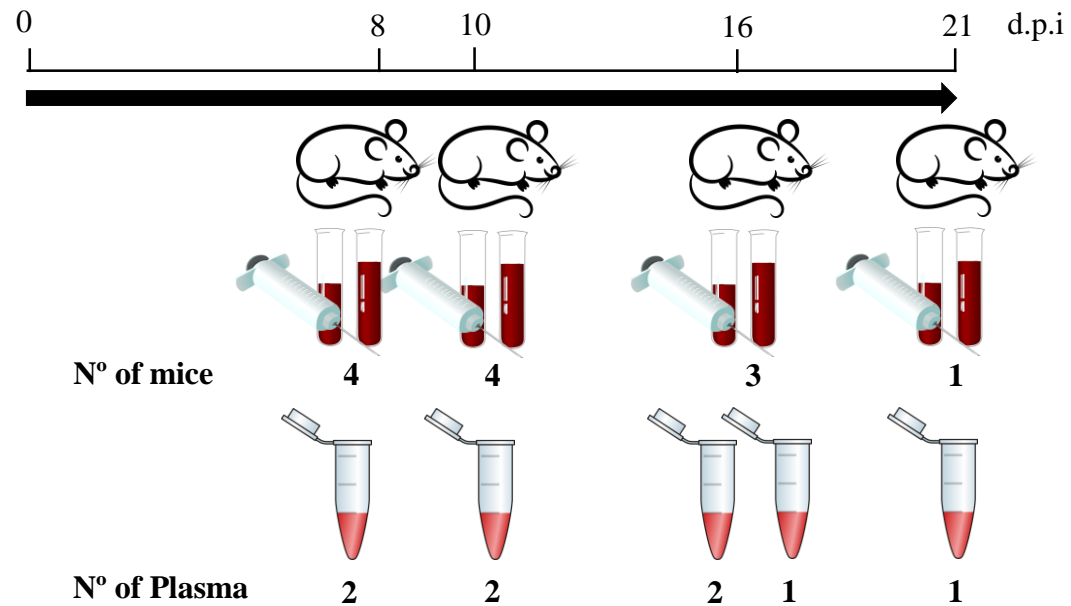

Supplement: Figure S1 — Experimental P. vivax infection of FRG huHep mice. (A) Six FRG huHep mice were infected with 1 million P. vivax sporozoites. Infected mice were euthanized 8 days post-infection (dpi) and blood samples collected. Four uninfected control mice were also euthanized and blood samples collected. (B) Twelve FRG huHep mice were infected with P. vivax sporozoites and euthanized 8 dpi (four mice), 10 dpi (four mice), 16 dpi (three mice), and 21 dpi (one mouse). After plasma was collected from individual mice, plasma samples from two mice were pooled to generate 2 plasma samples 8 dpi, 2 samples 10 dpi, and 2 samples 16 dpi (1 sample pooled from 2 mice and 1 sample from a single mouse). [file Image_1.PDF]
