# Supplementary material for: Characterization of Plasmodium vivax Proteins in Plasma-Derived Exosomes From Malaria-Infected Liver-Chimeric Humanized Mice
Source: Front Microbiol. 2018 Jun 25;9:1271. doi: 10.3389/fmicb.2018.01271 (PMC6026661; doi:10.3389/fmicb.2018.01271)
Supplement: Figure S2 — Molecular characterization of SEC fractions was done through flow cytometry bead-based analysis of the CD5L marker. (A) Experimental infection 1 (EI1). Isolation of exosomes from the plasma of P. vivax infected FRG HuHep mice was performed 8 days post infection (dpi). There were 6 infected mice (M1-M6) and 4 uninfected mice (M1-M4). (B) Experimental infection 2 (EI2). Isolation of exosomes from plasma of P. vivax infected FRG HuHep mice at 8 (2 mice: M1-M2), 10 (2 mice: M1-M2), 16 (2 mice: M1-M2), and 21 (1 mouse) dpi. Images show that CD5L-positive-exosomes were enriched in fractions 7−10. Based on this profile, an exosomal enriched fraction (ExEF) and a plasma-microvesicles enriched fraction (MvEF) were made by pooling fractions F7-F8-F9-F10 (black dashed square) and fractions F5-F6-F11-F12 (red dashed square), respectively. Blue circles: CD5L signal, Purple squares: negative control (Fractions+rabbit-isotype+Alexa488-2a antibody, Black diamonds: Protein concentration. [file Image_2.PDF]

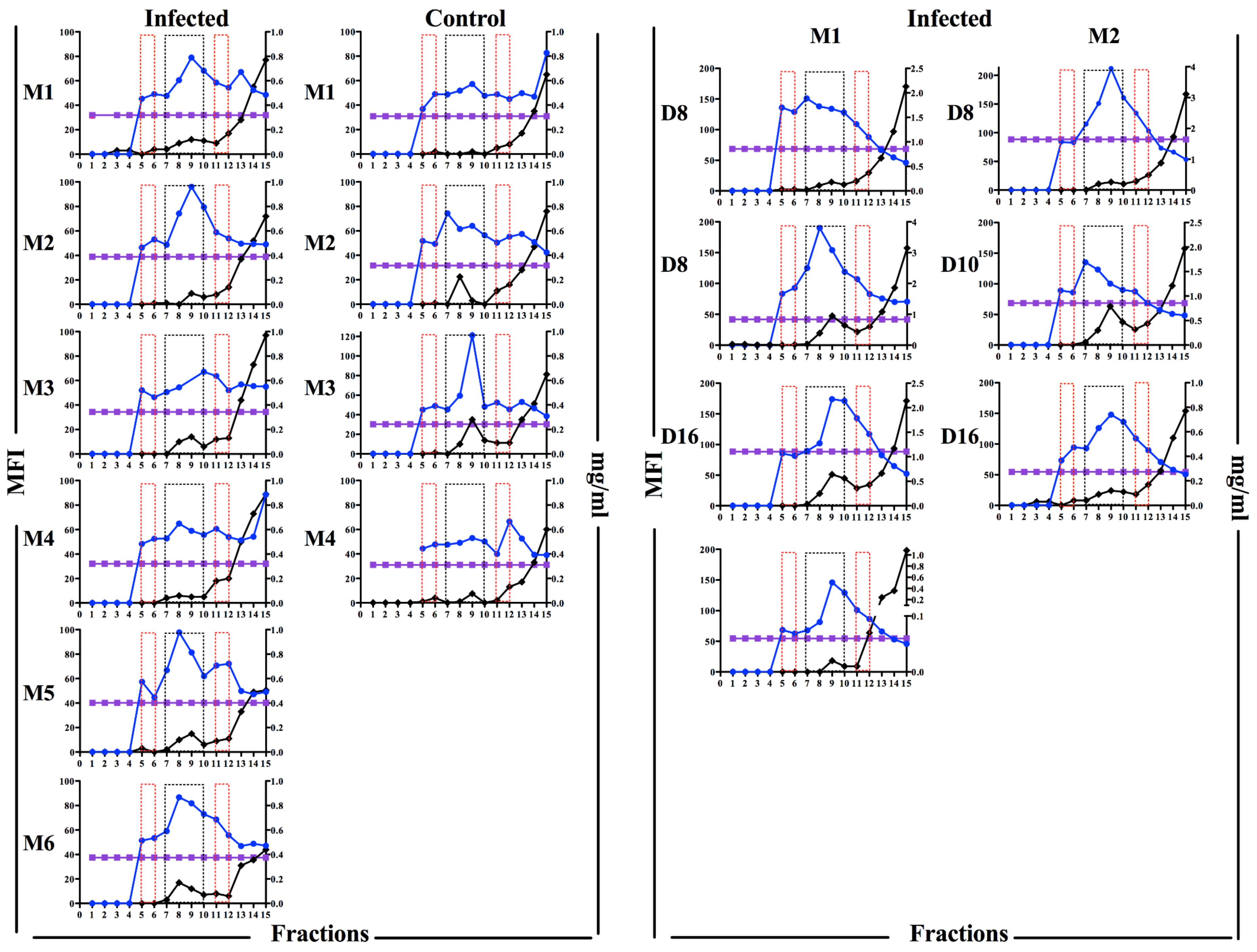

Infected
Control
M1
M2
M3
M4
M5
M6
MFI
mg/ml
Fractions
Infected
M1
M2
D8
D10
D16
MFI
mg/ml
Fractions
